# Washing of the Stomach

**Published:** 1882-07-20

**Authors:** Austin C. Ramsby

**Affiliations:** Chicago


### WASHING OF THE STOMACH.

**Translated for the Chicago Medical Journal and Examiner**

BY AUSTIN C. RAMSBY, CHICAGO.

*Washing of the Stomach.*—With the perfection and simplicity of the instrumental outfit, the washing of the stomach is going to take an important part in the practice of medicine. It constitutes a precious resource against affections of the stomach, and all the physicians who have tried it unanimously report happy results.

The instruments for that operation are all well known. The pump of Kusslmaul, the Dujardin-Beaumetz instrument, the Siphon-tube of Fancher, have all been presented to the readers of the Revue Medicale. Dr. Fancher's instrument seems to be the best, for two reasons: 1st. The facility of its introduction, which even the patient can do himself after a few operations. 2d. Because most everywhere it can be improvised—for a piece of rubber tubing of 1.50 long and 10 to 12 millimeters in diameter can almost always be had, and every household has a common funnel which can be used. Before speaking of the therapeutical indications for the washing of the stomach, we will describe the operation:

The patient should be sitting, the head looking directly forward at almost a right angle with the vertebral column. Some physicians prefer reversing the head backward, in order to introduce the sound vertically.

For pusillanimous patients the dorsal decubitus might offer certain advantages. In the first operation, to establish the habit and overcome the spasms of the pharynx, the use of the rigid sound is preferable; the common œsophagial sound answers the purpose well. But when, after repeating the operation a few times the patient knows how to introduce the rigid sound without pushing it, but by the simple action of deglutition, then the common red rubber tube must be substituted for the sound. The tube must be well oiled. The tube for females ought to be of smaller caliber than for males. A mark on the tube at 45 or 50 centimeters from its free extremity will indicate, when touching the lips, that the stomach has been reached. But this generally is known by a spasm of the cardia, which react to a slight pressure and causes an effort to vomit. Sometimes, but rarely, there is a sudden escape of gases, and of the contents of the stomach; this must be borne in mind in order to protect the patient's clothing. To fill the tube the funnel must be raised one meter above the level of the stomach, and filled with liquid to the amount of from one to two liters; then, by bringing it below the epigastric region, the fluid injected can be seen filling the funnel and holding in suspension the matter contained in the stomach. Dr. Constantin Paul advises the use of cold alkaline water, terminating the operation with warm water. The antiseptics, etc., are highly spoken of.

The operation terminates with the introduction of from 2 to 300 grams of milk in the stomach, and, to prevent the pyloric dilatation, the abdomen must be compressed with a large bandage, or, when possible, have the patient walk for a few minutes.

Such are the details of the operation according to Dr. Paul. We will see now what benefits we can derive from its use.

The washing of the stomach will instantly suppress the pains in cases of neuralgia and chronic gastritis; it will calm the consecutive oppression in cases of dilatation of the stomach.

It stimulates the appetite, and brings a rapid augmentation in weight. Drs. See, Buocquoy, Ferrand and Paul, have had surprising results from it in cases of gastritis, hysterical vomiting, dyspepsia, ulcers and cancers of the stomach; in this last, it is true, the relief is only temporary. Dr Paul has even utilized it in a case of strangulated hernia, to save the patient the disagreeableness of fœcal vomiting.

Its use in cases of recent poisoning need not be commented on. —*Revue Medical.—Chicago Med. Jour.*

